# Nanoscale MoS_2_-in-Nanoporous Au Hybrid Structure for Enhancing Electrochemical Sensing

**DOI:** 10.3390/s25237137

**Published:** 2025-11-22

**Authors:** Jihee Kim, Minju Kim, Yunju Choi, Jong-Seong Bae, Seunghun Lee, Robert A. Taylor, Andy Chong, Kwangseuk Kyhm, Mijeong Kang

**Affiliations:** 1 Department of Cogno-Mechatronics Engineering, Pusan National University, Busan 46241, Republic of Korea; 2 Yeongnam Regional Center, Korea Basic Science Institute, Busan 46742, Republic of Korea; 3 Department of Physics, Pukyong National University, Busan 48513, Republic of Korea; 4 Clarendon Laboratory, University of Oxford, Parks Road, Oxford OX1 3PU, UK; 5 Department of Physics, Pusan National University, Busan 46241, Republic of Korea; 6 Institute for Future Earth, Pusan National University, Busan 46241, Republic of Korea; 7 Crystal Bank Research Institute, Pusan National University, Busan 46241, Republic of Korea

**Keywords:** transition metal dichalcogenides, nanoporous structure, nanoconfinement effect, redox cycling reaction, electrochemical sensor

## Abstract

We report the fabrication of nanoscale MoS_2_ (nMoS_2_) via laser ablation in liquid and its application in electrochemical sensing. The laser ablation process fragments microscale MoS_2_ sheets into ~5 nm dots with stable aqueous dispersibility. Electrochemical analysis reveals that nMoS_2_ possesses multiple reversible redox states, enabling it to participate in redox cycling reactions that can amplify electrochemical signals. When the nMoS_2_ is embedded in an electrochemically inert matrix, a chitosan layer, and subsequently incorporated within a nanostructured Au electrode, the nMoS_2_-participating redox cycling reactions are further enhanced by the nanoconfinement effect, leading to synergistic signal amplification. As a model system, this hybrid nMoS_2_-in-nanoporous Au electrode demonstrates a 9-fold increase in sensitivity for detecting pyocyanin, a biomarker of *Pseudomonas aeruginosa* infection, compared with a flat electrode without nMoS_2_ loading. This study not only elucidates the redox characteristics of laser-fabricated zero-dimensional transition metal dichalcogenides but also presents a strategy to integrate semiconducting nanomaterials with metallic nanostructures for high-performance electrochemical sensing.

## 1. Introduction

Transition metal dichalcogenides (TMDs) are two-dimensional chalcogenide–transition metal–chalcogenide trilayer materials that possess electronic structures distinct from their bulk counterparts, making them highly attractive for various applications such as semiconductors, energy devices, and catalysis. In their pristine state, TMDs form layered bulk bound by van der Waals interaction between individual monolayers, and as the number of layers decreases, the energy of the out-of-plane orbitals is affected, inducing changes in the electronic structure—for instance, a transition from an indirect to a direct bandgap [1]. When monolayer TMDs are used in solution, however, van der Waals interaction causes restacking [2] and limits their applications. By fragmenting the monolayer TMDs into nanoscale pieces with lateral dimensions of a few nanometers (i.e., forming zero-dimensional TMDs), the dispersibility can be improved and therefore their usability in solutions can be enhanced [3]. Furthermore, additional modifications in the electronic structure may occur due to quantum confinement effects, defects, and charge accumulation associated with size reduction [4]. In this study, we prepare nanoscale MoS_2_, a representative TMD, and demonstrate a method to utilize its unique electronic structure and the related redox behavior including its capability to act as an electron reservoir in aqueous solutions. MoS_2_ has been employed in various solution-phase applications, and we specifically focus on nanoscale-MoS_2_-based electrochemical sensing. Nanoscale MoS_2_ is used as electrocatalysts to amplify electrochemical signals, although the target substances are rather limited (e.g., H_2_O_2_) [5]. To fully exploit the potential of nanoscale MoS_2_ in electrochemical sensing, it is necessary to uncover its unexplored properties and develop new sensing mechanisms based on them.

Among various methods to fabricate nanoscale TMDs, laser ablation in liquid has attracted substantial interest. Laser irradiation generates plasma and cavitation bubbles around bulk TMDs, where high temperature, pressure, and shock waves are involved [6,7]. In addition, laser irradiation was known to induce structural disruption of TMD sheets, followed by rapid cooling and condensation in the liquid to fragment the TMD sheets. Laser ablation in liquid (i) requires no additives and thereby no post-treatments to obtain pure zero-dimensional TMDs, (ii) operates under ambient conditions, providing a green fabrication route, and (iii) owing to the wide tunability of laser and liquid parameters, allows versatile control over the characteristics of the resulting zero-dimensional TMDs. Fabrication of nanoscale MoS_2_ by laser ablation has also been reported, yielding brownish solutions containing few-nanometer MoS_2_ dots [8,9,10].

**Figure 1:**
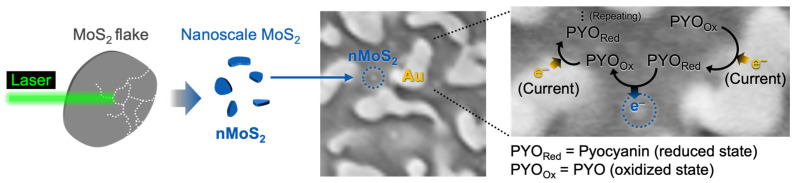
Schematic representation of preparing nMoS_2_ and its application to improve the electrochemical detection of the bacterial infection biomarker (pyocyanin).

In this study, we fabricated nanoscale MoS_2_ (nMoS_2_) via laser ablation in liquid and used its unique electronic structure to enhance the performance of electrochemical sensing as a model application (Figure 1). Our group has previously reported the amplification of electrochemical signals based on redox cycling reactions with MoS_2_ flakes having a lateral size of a few micrometers [11]. nMoS_2_ can provide an additional signal amplification strategy by utilizing the nanoconfinement effect in the sensing process. Within a space confined at nanoscale, the electrochemical reaction rate can be increased (that is, the nanoconfinement effect), which can dramatically facilitate redox cycling reactions (note: the details are discussed in Results and Discussion) [12]. For the nanoconfinement effect with MoS_2_, it is essential to place MoS_2_ within a nanostructured space, and we successfully fabricated such a nMoS_2_-in-nanoporous Au hybrid structure. nMoS_2_ was synthesized via laser ablation by a 532 nm nanosecond pulse laser, and its morphology, optical characteristics, and redox behavior were analyzed. nMoS_2_ was subsequently integrated onto the surface of nanostructured electrodes prepared by an alloying–dealloying process, leading to the amplification of redox cycling-assisted electrochemical signals. Finally, as a model application of electrochemical sensing, we utilized nMoS_2_-in-nanoporous Au to enhance the electrochemical signal of pyocyanin, a biomarker of *Pseudomonas aeruginosa* infection.

## 2. Materials and Methods

*Preparation of nMoS_2_.* First, MoS_2_ suspension was prepared with MoS_2_ flakes (particle size < 5 μm) in deionized water (DIW, 18.2 MΩ·cm at 25 °C) at a concentration of 1 g per 10 mL. The ablation of MoS_2_ flakes was carried out using an AOM Q-switched 532 nm laser emitting nanosecond pulses with a pulse duration of 0.5 ns and a repetition rate of 1 kHz. The collimated laser beam was introduced to a glass vial containing the MoS_2_ suspension, positioned to ensure the interaction with MoS_2_ flakes for 24 h at a power of 500 mW. The suspension was continuously stirred during the ablation process to effectively expose the MoS_2_ flakes to the laser. Following the ablation process, the suspension was allowed to precipitate undisturbed for 2 h and then centrifuged at 10,000 rpm for 20 min to isolate the fragmented nMoS_2_. The supernatant containing nMoS_2_ was collected and used in subsequent experiments. To observe the morphology of nMoS_2_, transmission electron microscopy (TEM) analysis was performed using a JEOL JEM-2100F (Instrument code: SU09) at the KBSI Yeongnam Regional Center.

*Preparation of nanoporous Au electrodes.* A nanostructured Au layer was fabricated on a flat Au electrode by using the typical alloying–dealloying process [13]. First, a flat Au electrode was cleaned by ultrasonication and then standard electrochemical cleaning [14]. Second, the AuAg alloy was electrodeposited to the flat Au electrode in an aqueous solution of 6 mM KAu(CN)_2_, 24 mM KAg(CN)_2_, and 50 mM Na_2_CO_3_ at −1.2 V (vs. Ag/AgCl) for 30 s. From the AuAg alloy, Ag was dissolved away in nitric acid, leaving a layer of nanoporous Au.

*Modification of electrode surface with nMoS_2_.* The Au electrode was first coated with a compact monolayer of mercaptohexanol (MCH) in an aqueous solution of 2 mM MCH. An acidic solution of chitosan containing nMoS_2_ was prepared by mixing a 1.2 wt% chitosan solution and a nMoS_2_ solution at 2:1 ratio. This solution was drop-cast on the MCH-coated Au electrode, followed by neutralization with phosphate buffer (PB) to induce the sol–gel transition of chitosan. In this way, nMoS_2_ was embedded in an electrochemically inactive chitosan layer formed on an Au electrode.

*Electrochemical measurements.* Electrochemical experiments were conducted using a three-electrode system of an Au working electrode, a Ag/AgCl (3 M KCl) reference electrode, and a Pt wire counter electrode with a potentiostat (CH Instruments, Autin, TX, USA). Cyclic voltammetry (CV) was conducted at a scan rate of 0.1 V/s in a PB solution (0.1 M, pH 7.2) without deaeration.

## 3. Results and Discussion

### 3.1. Morphological Properties of nMoS_2_

**Figure 2:**
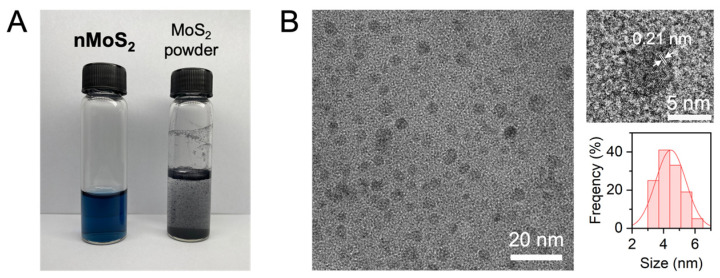
Aqueous solution of nMoS_2_. (**A**) An nMoS_2_ solution in deionized water (DIW) and a suspension of MoS_2_ powder in DIW. (**B**) Transmission electron microscopy images of nMoS_2_ and particle size distribution.

Figure 2A shows the aqueous solution of nMoS_2_ fragmented from microscale MoS_2_ via laser ablation in liquid. Unlike the suspension of microscale MoS_2_, which appears opaque and grayish, the nMoS_2_ solution is transparent with a bluish color. Previously reported nanoscale MoS_2_ prepared by laser ablation appeared yellowish in color [8,9,10]. This difference in the solution color is attributable to the different characteristics of laser and liquid. For instance, the nanoscale MoS_2_ in Ref [9] was prepared in water with a femtosecond 800 nm laser at 3.5 mW; the laser parameters differ from those used in this study. To understand the effect of laser parameters on the fragmentation, a detailed systematic study is required.

The bluish color of our nMoS_2_ solution remained stable when stored at 4 °C. The TEM image in Figure 2B reveals the centrosymmetric morphology of nMoS_2_ with a size of 4.5 ± 1.0 nm. From the high-resolution TEM image, the lattice fringe of 0.20 nm is observed, which is ascribed to the (006) plane of MoS_2_. In addition to this nanoscale dimension, the surface charge characteristics enable nMoS_2_ to remain stably dispersed in an aqueous solution. nMoS_2_ exhibits a zeta potential of ca. −28 mV, which indicates its good stability in water [15]. This is attributable to sulfur vacancies, the most dominant defect in MoS_2_, acting as n-type dopants [16]. Sulfur vacancies are thermodynamically more favorable at the edges than on the basal plane [17]. Thus, a larger fraction of edge sites in nMoS_2_ is expected to significantly influence the surface charge of nMoS_2_ by introducing an increased density of sulfur vacancies and improves its dispersibility in an aqueous solution.

### 3.2. Optical Properties of nMoS_2_

**Figure 3:**
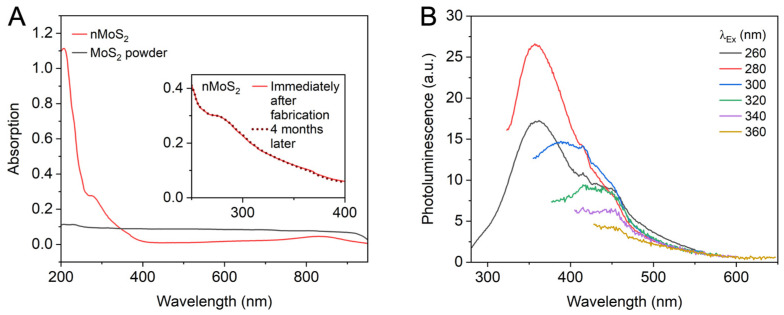
Optical properties of nMoS_2_. (**A**) UV-Vis absorption spectra of the nMoS_2_ aqueous solution (red) and the MoS_2_ powder suspension in DIW (black). Inset: UV-Vis absorption spectra of nMoS_2_ immediately after fabrication and after four months. (**B**) Photoluminescence spectra of nMoS_2_ with varying excitation wavelengths (λ_Ex_).

The size of nMoS_2_ substantially influences its optical properties. We observed the absorption spectra of nMoS_2_ and its microscale counterpart, MoS_2_ flakes (Figure 3A). While microscale MoS_2_ absorbs broadly across the visible range, nMoS_2_ displays a distinct peak in the UV region (at 276 nm) [9,18,19,20]. This feature originates from the excitonic behavior of nMoS_2_, which becomes pronounced when the material size approaches the excitonic Bohr radius of MoS_2_ (1.61 nm [21]). Thus, Figure 3A confirms the nanoscale dimension of nMoS_2_. [Note: the inset of Figure 3A shows the UV-Vis absorption spectra of the nMoS_2_ aqueous solution measured immediately after fabrication and four months later, with no discernible change observed. This indicates that the nMoS_2_ aqueous solution is stable for at least four months]. Additional size-related information can be obtained from photoluminescence measurements. When MoS_2_ monolayers are exfoliated from the bulk, their electronic structures are altered resulting in the transition from an indirect to a direct bandgap, consequently generating photoluminescence (PL) as shown in Figure 3B. The emission peak of nMoS_2_ is red-shifted as the excitation wavelength changes from 260 to 360 nm. The maximum PL intensity is observed when the excitation wavelength is set close to the absorption peak. This wavelength-dependent photoluminescence can be attributed to the polydispersity of nMoS_2_ [22,23].

### 3.3. Redox Properties and Redox Cycling-Assisted Signal Amplification of nMoS_2_

**Figure 4:**
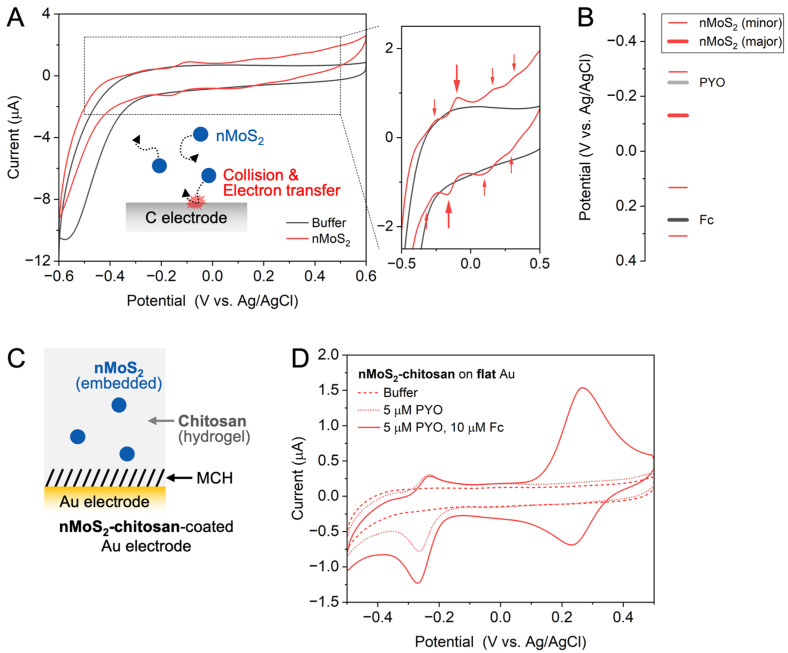
Redox properties of nMoS_2_. (**A**) Cyclic voltammograms of a phosphate-buffer (PB) solution before (black) and after (red) adding nMoS_2_ to the solution. (**B**) Thermodynamic plot showing the redox potentials of nMoS_2_ (red), pyocyanin (PYO, light gray) and ferrocenedimethanol (Fc, dark gray). (**C**) Schematic representation of the nMoS_2_-chitosan-coated Au electrode where a self-assembled monolayer of mercaptohexanol (MCH) is formed before the nMoS_2_-chitosan coat. (**D**) Cyclic voltammograms obtained from nMoS_2_-chitosan-coated flat Au electrode in a PB solution before (dashed) and after adding PYO (dotted) and Fc (solid).

To investigate the electrochemical applicability of nMoS_2_, we examined its intrinsic redox properties. As shown in Figure 4A, cyclic voltammetry (CV) was employed to identify the redox states of nMoS_2_. Cyclic voltammograms were recorded in a PB solution using a glassy carbon electrode, and the difference between the voltammograms obtained before and after adding nMoS_2_ reveals its intrinsic redox behavior. In the voltammogram of the nMoS_2_ solution from 0 V (vs. Ag/AgCl) toward negative potentials, a relatively strong peak and a weaker reduction peak appear at −0.16 V and −0.32 V, respectively. Beyond these potentials, large reduction currents are observed, which can be attributed to the reduction of O_2_ (that is, oxygen reduction reaction, ORR) dissolved in the solution, as also observed in the cyclic voltammogram of a PB solution without nMoS_2_. When the potential scan is reversed, oxidation peaks appear at −0.26 V and −0.10 V, corresponding to the oxidation of the reduced nMoS_2_ species produced at −0.32 V and −0.16 V, respectively. Each pair of reduction and oxidation peaks exhibit comparable peak currents, indicating the reversibility of these redox processes. From these two pairs of redox peaks, the formal reduction potentials of the two redox states of nMoS_2_ are determined to be approximately −0.29 V and −0.13 V. When the potential is scanned further in the positive direction, additional oxidation peaks appear at +0.16 V and +0.33 V, and upon reversing the scan again, corresponding reduction peaks are observed at +0.28 V and +0.10 V, defining two additional redox states of the formal reduction potentials at +0.30 V and +0.13 V, respectively. The thermodynamic plot in Figure 4B shows the energy levels of these four distinct redox states of nMoS_2_. The redox states of nMoS_2_ significantly differ from that of microscale MoS_2_ reported previously [11], which exhibited only a single redox state around +0.24 V. The single redox state of microscale MoS_2_ was attributed to an in-gap state, presumably arising from intrinsic structural defects of MoS_2_. nMoS_2_ is expected to possess a variety of structural defects generated during the fragmentation process [6], along with a significantly increased fraction of edges that have electronic characteristics distinct from the basal plane [24]. In this way, the formation of additional in-gap states of nMoS_2_ is expected, giving rise to the multiple redox states. Based on the peak currents, it is expected that the redox state around −0.13 V (thick red line in Figure 4B) plays a major role in the redox behavior of nMoS_2_.

**Figure 5:**
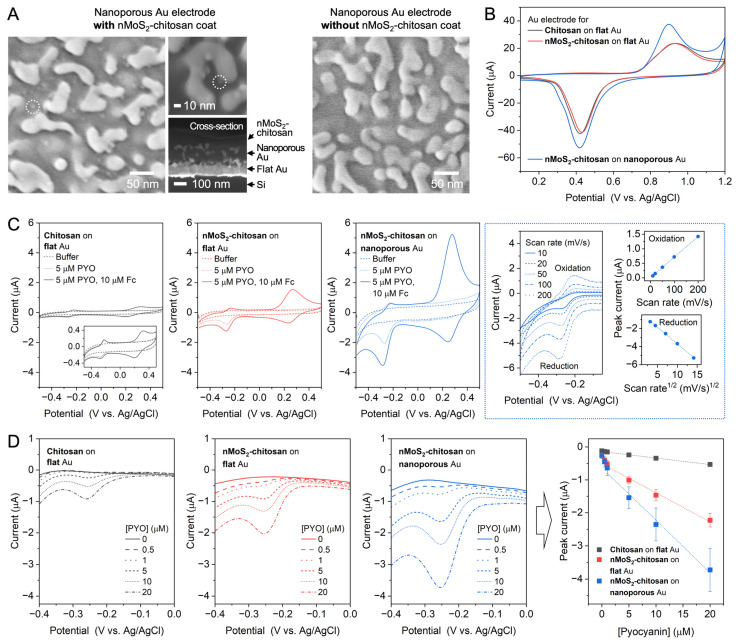
nMoS_2_ in nanoporous Au for nanoconfinement-assisted amplification of sensing signals. (**A**) Scanning electron microscopy images of nanoporous Au electrode with (**left**) and without (**right**) nMoS_2_-chitosan coat. Dotted circles in the left image indicate the presence of nMoS_2_ within the nanoporous Au. (**B**) Cyclic voltammograms for the oxidation and reduction in Au electrodes. (**C**) Cyclic voltammograms obtained from chitosan-coated flat Au electrode, nMoS_2_-chitosan-coated flat Au electrode, and nMoS_2_-chitosan-coated nanoporous Au electrode in a phosphate-buffered solution before (dashed) and after adding pyocyanin (PYO, dotted) and ferrocenedimethanol (Fc, solid). The rightmost panels in the dotted rectangle show the scan-rate-dependence of the PYO reduction peak current (proportional to the square root of the scan rate) and PYO oxidation peak current (proportional to the scan rate). (**D**) PYO reduction currents obtained from chitosan-coated flat Au electrode, nMoS_2_-chitosan-coated flat Au electrode, and nMoS_2_-chitosan-coated nanoporous Au electrode with increasing the PYO concentrations in the presence of 50 μM Fc. The rightmost panel shows the peak currents of PYO reduction as a function of PYO concentration with two linear fit curves for each electrode group.

The thermodynamic plot in Figure 4B includes not only the redox states of nMoS_2_ but also those of two other species: pyocyanin (PYO), which is the target analyte to be electrochemically detected, and ferrocenedimethanol (Fc), which maximizes the capability of nMoS_2_ in detecting PYO. PYO initially exists in an electron-deficient state and is reduced at the electrode at potentials more negative than approximately −0.2 V. When the electron-rich (i.e., reduced at an electrode) PYO encounters electron-deficient (i.e., oxidized) nMoS_2_, it can transfer its electron to nMoS_2_ and be subsequently reduced again at the electrode, resulting in an amplification of the PYO reduction current. For this electron transfer to occur, two conditions must be satisfied: (i) redox-active nMoS_2_ must be electrically isolated from the electrode, enabling it to accept electrons directly from PYO rather than the electrode, and (ii) nMoS_2_ must possess a redox state more positive than that of PYO. The thermodynamic plot in Figure 4B confirms that the latter condition is indeed met; the principal redox state of nMoS_2_ is located 0.12 V more positive than the standard reduction potential of PYO. To achieve the former, we embedded nMoS_2_ within an electrochemically inert matrix (here, a chitosan layer [25] as illustrated in Figure 4C) coated on the electrode surface, which will be hereafter denoted as nMoS_2_-chitosan (note: electrode surface had been modified with MCH before nMoS_2_-chitosan to prevent ORR). Recorded from this nMoS_2_-chitosan-coated electrode (Figure 4D), the cyclic voltammogram obtained in buffer solution shows no observable redox signals of nMoS_2_, confirming that nMoS_2_ is electrically isolated from the electrode within the chitosan matrix. In this structure, nMoS_2_ can enhance its capability to accept electrons from PYO by first transferring its own electrons to another redox-active species of which the redox state resides at a more positive potential range than that of nMoS_2_; this is achieved by adding Fc to this electrochemical system (see the thermodynamic plot in Figure 4B). After Fc is oxidized at an electrode in the positive potential scan, it returns to its reduced state by depriving electrons from nMoS_2_ and gets oxidized again at an electrode; this redox cycling repeats continuously. As a result, nMoS_2_ gradually becomes more electron-deficient, thereby increasing its capacity to accept electrons from the reduced PYO during the negative potential scan. This is clearly demonstrated in Figure 4D, where the PYO reduction current increases significantly after the addition of Fc to the solution although Fc itself is inactive in the potential range of the PYO signal. Compared with the control electrode coated only with chitosan (without nMoS_2_), the nMoS_2_-chitosan-coated electrode amplifies the PYO signal by approximately 4.3-fold, which will be further discussed in Figure 5. Overall, Figure 4 illustrates that nMoS_2_ possesses suitable redox states for the electrochemical detection of PYO, and that in the presence of an appropriate oxidizing agent such as Fc, the detection signal can be further amplified.

### 3.4. Nanoconfinement Effect-Assisted Signal Amplification by nMoS_2_

To exploit the nanoscale characteristics of nMoS_2_ for enhancing its performance in electrochemical sensing, we employed the nanoconfinement effect. The nanoconfinement effect refers to the increased frequency of the collision between electroactive molecules and the surface of a nanostructured electrode once the molecule enters the interior of the electrode, thereby increasing the probability of electron exchange between them [26]. When nMoS_2_ placed near the electrode surface and the freely diffusing redox molecules PYO and Fc coexist within the nanostructured electrode, the redox cycling reactions between nMoS_2_, PYO, and the electrode, and between nMoS_2_, Fc, and the electrode would be facilitated due to the nanoconfinement effect. As a consequence, the electrochemical reduction in PYO can be significantly amplified. To fabricate such a nanostructured Au electrode, we employed a well-established alloying–dealloying process [13]. A nanoporous Au electrode with a pore size of a few tens of nm was obtained by electrochemically co-depositing Au and Ag, followed by chemical removal of Ag, as shown in Figure 5A. The formation of nanoporous structure was also confirmed through an increase in the electrochemically active surface area. Figure 5B shows the cyclic voltammograms for the oxidation and reduction in the outermost Au layer of the flat Au electrodes and the nanoporous Au electrode. By comparing the charge of the reduction peak around +0.4 V, it is found that the surface area of nanoporous Au is approximately 1.3-fold larger than that of flat Au.

Onto the nanoporous Au electrode, we formed the nMoS_2_-chitosan layer and performed CV in the presence of PYO and (Figure 5C). As shown in the scanning electron microscopy images of the surface and cross-section of the nMoS_2_-chitosan-coated nanoporous electrode (Figure 5A), the pore was filled with the nMoS_2_-chitosan layer, successfully placing the nMoS_2_ inside the nanoscale space of the electrode. PYO reduction is approximately 2.9- and 12.3-fold larger when it is recorded by the nMoS_2_-chitosan-coated electrode than the flat electrode with the nMoS_2_-chitosan layer and the flat electrode with the chitosan layer, respectively (Figure 5C). [Note: PYO oxidation is not influenced by the properties of the electrode as much as PYO reduction. A detailed explanation is provided below]. It is clearly seen that nMoS_2_ itself amplifies the PYO reduction (i.e., chitosan-coated flat Au vs. nMoS_2_-chitosan-coated flat Au), and nanoporous Au assists the amplification further. Considering that the surface area of the nanoporous Au electrode is only about 1.3 times larger than that of the flat Au electrode, it is concluded that the amplification of PYO reduction by the nanoporous Au electrode arises not only from the increased surface area but also from the nanoconfinement effect. Thus, by employing this hybrid system, the nanoscale redox-active semiconducting materials confined within a nanostructured metallic electrode, the electrochemical signal can be maximized through synergistic amplification.

Figure 5C also shows the kinetic aspect of the nMoS_2_-associated redox-cycling reaction. We obtained cyclic voltammograms at different scan rates and analyzed the scan-rate dependence of peak currents. The peak current of PYO oxidation is linearly dependent on the scan rate while that of PYO reduction is linearly dependent on the square root of the scan rate. To interpret these scan-rate dependencies, it is important to note that, unlike PYO reduction, PYO oxidation is not affected by the nMoS_2_-associated redox-cycling reaction. During the PYO reduction, PYO is reduced at the electrode and then can be oxidized when it diffuses and encounters the nMoS_2_ in the oxidized state, repeatedly being reduced (amplifying reduction current) and oxidized (yielding no current); in this process, nMoS_2_ is charged. During the PYO oxidation, PYO is oxidized at the electrode, but it cannot be reduced by the charged nMoS_2_, which is essential for the nMoS_2_-associated redox-cycling reaction, because of the relative potential levels of their redox states. For this PYO oxidation, the nanoporous Au electrode can behave as a thin layer cell [27]; the characteristic pore dimensions are much smaller than the typical diffusion lengths of small molecules, which are in micrometer scale, therefore limiting the dimension of the diffusion layer. Under this condition, the PYO oxidation peak current is dependent on the scan rate, as observed in Figure 5C. In contrast, the PYO reduction peak current is proportional to the square root of the scan rate, which is a characteristic of the diffusion-controlled reaction under the condition of semi-infinite linear diffusion. This indicates that the nMoS_2_-associated redox-cycling reaction is fast enough, continuously supplying regenerated oxidized PYO and creates an effect as if the diffusion layer is expanded.

The nMoS_2_-in-nanoporous Au electrode hybrid system provides increased sensitivity in detecting PYO as demonstrated in Figure 5D. Cyclic voltammograms in Figure 5D were recorded from chitosan-coated flat Au electrode, nMoS_2_-chitosan-coated flat Au electrode, and nMoS_2_-chitosan-coated nanoporous Au electrode while varying the PYO concentration in the presence of excess Fc (to ensure the maximum capacity of nMoS_2_ in amplifying PYO reduction currents). The PYO reduction peak currents as a function of PYO concentration are also displayed (N = 3 for each electrode). Due to the nonlinearity observed across the entire concentration range, the calibration curve was segmented into two linear response regions at low (0–1 μM) and high (1–20 μM) concentrations. From the calibration curves in the low concentration range, the sensitivities are calculated to be 0.4 × 10^−7^, 2.6 × 10^−7^, and 3.7 × 10^−7^ μA·μM^−1^ and the limit of detection to be 3.8, 1.6, and 1.4 μM (calculated by the general 3σ method) for chitosan-coated flat Au, nMoS_2_-chitosan-coated flat Au, and nMoS_2_-chitosan-coated nanoporous Au electrodes, respectively; the combination of nMoS_2_ and nanoconfinement effect leads to a 9-fold enhancement in detection sensitivity. This model application demonstrates that nMoS_2_ can improve the performance of electrochemical sensing, particularly through synergistic effects with nanostructured metallic electrodes.

## 4. Conclusions

In summary, we have prepared nanoscale MoS_2_ (nMoS_2_) via laser ablation in liquid and elucidated its redox characteristics for utilization in electrochemical sensing. Using cyclic voltammetry, we reveal that the zero-dimensional nMoS_2_ has multiple reversible redox states, providing a versatile platform for a redox cycling reaction that can be used for the amplification of electrochemical signals. Interestingly, when incorporated within nanostructured Au electrodes, the nMoS_2_-participating redox cycling reaction is facilitated via nanoconfinement effect, leading to additional signal amplification. This hybrid configuration of nanoscale semiconducting material-in-nanostructured metallic material improves the sensitivity of electrochemically detecting the bacterial infection biomarker by nine times. There are opportunities for achieving further improvement in sensor performances by optimizing this system in three different aspects; (i) combinations of laser parameters (e.g., wavelength, pulse width, power) and liquid properties (e.g., polarity, viscosity, temperature) in the process of preparing nMoS_2_ by laser ablation in liquid, (ii) structural properties (e.g., pore size, pore shape, thickness) of nanostructured electrodes, and (iii) surface functionalization of the nanostructured electrode with nMoS_2_ (e.g., embedding in matrix other than chitosan, depositing at a monolayer level, covalently tethering). We believe this study opens a new way for preparing and utilizing zero-dimensional TMDs for advanced electrochemical applications.

## Data Availability

The data supporting the findings of this study are available within the article. Additional data that support the findings of this study are available from the corresponding author upon reasonable request.
